# Immunogenic SARS-CoV-2 S and N Protein Peptide and Cytokine Combinations as Biomarkers for Early Prediction of Fatal COVID-19

**DOI:** 10.3389/fimmu.2022.830715

**Published:** 2022-03-21

**Authors:** Ekaterina Martynova, Shaimaa Hamza, Maria Markelova, Ekaterina Garanina, Yuriy Davidyuk, Venera Shakirova, Neha Kaushal, Manoj Baranwal, Robert J. Stott-Marshall, Toshana L. Foster, Albert Rizvanov, Svetlana Khaiboullina

**Affiliations:** ^1^Institute of Fundamental Medicine and Biology, Kazan Federal University, Kazan, Russia; ^2^Department of Infectious Diseases, Kazan State Medical Academy, Kazan, Russia; ^3^Department of Biotechnology, Thapar Institute of Engineering and Technology, Patiala, India; ^4^Faculty of Medicine and Health Sciences, School of Veterinary Medicine and Science, Wolfson Centre for Global Virus Research, University of Nottingham, Loughborough, United Kingdom

**Keywords:** peptide, COVID-19, SARS-CoV-2, fatal, cytokine

## Abstract

Early indications of the likelihood of severe coronavirus disease 2019 COVID-19 can influence treatments and could improve clinical outcomes. However, knowledge on the prediction markers of COVID-19 fatality risks remains limited. Here, we analyzed and quantified the reactivity of serum samples from acute (non-fatal and fatal) and convalescent COVID-19 patients with the spike surface glycoprotein (S protein) and nucleocapsid phosphoprotein (N protein) SARS-CoV-2 peptide libraries. Cytokine activation was also analyzed. We demonstrated that IgM from fatal COVID-19 serum reacted with several N protein peptides. In contrast, IgM from non-fatal serum reacted more with S protein peptides. Further, higher levels of pro-inflammatory cytokines were found in fatal COVID-19 serum compared to non-fatal. Many of these cytokines were pro-inflammatory and chemokines. Differences in IgG reactivity from fatal and non-fatal COVID-19 sera were also demonstrated. Additionally, the longitudinal analysis of IgG reactivity with SARS-CoV-2 S and N protein identified peptides with the highest longevity in humoral immune response. Finally, using IgM antibody reactivity with S and N SARS-CoV-2 peptides and selected cytokines, we have identified a panel of biomarkers specific to patients with a higher risk of fatal COVID-19 compared with that of patients who survive. This panel could be used for the early prediction of COVID-19 fatality risk.

## Introduction

A local outbreak of a severe pneumonia of unknown etiology in Wuhan, China, spread rapidly and was declared a pandemic in 2020; since then, there have been hundreds of millions of cases and over four million deaths worldwide (1). A novel member of the beta-coronavirus family, severe acute respiratory syndrome coronavirus-2 (SARS-CoV-2) was isolated early during the outbreak and it was found to cause coronavirus-induced disease (COVID)-19 (2). A large proportion of COVID-19 cases are asymptomatic, while disease severity is mostly linked with cases in older patients and those with underlying conditions (3–6). Infection is characterized by early activation of humoral immune responses where IgM and IgG peak at week five of the disease (7). Conversely, Iyer et al. have shown that IgM, IgG, and IgA levels reach the highest levels between 14 and 28 days followed by a gradual decline (8). SARS-CoV-2 Spike (S) and nucleocapsid (N) proteins have been identified as major immunogens (9) with IgG antibodies against the N and S proteins detected at the same time, supporting their highly immunogenic status (10).

Anti-SARS-CoV-2 antibodies contribute to severity and recovery from COVID-19. Sun et al. reported high anti-S protein IgG antibodies in non-intensive care unit (ICU) patients, while high anti-N protein IgG antibodies have been found in ICU patients (9). In addition, Röltgen et al. demonstrated a higher ratio of anti-S IgG/anti-N IgG antibodies in outpatients with mild COVID-19 (11). These data suggest differences in the antibody immune response to SARS-CoV-2 which may contribute to differences in severity of COVID-19. However, there is limited knowledge on how reactivity with SARS-CoV-2 S and N protein peptides differs between COVID-19 patients who require ICU treatment and those with only mild COVID-19.

Multiple S and N protein epitopes have been identified through COVID-19 patient serum reactivity studies conducted globally, including in China and the United States 12–14). These data will help to determine common peptides in the immune response to SARS-CoV-2 around the world. Upon identification of immunogenic regions of S and N proteins, they can be used to design subunit vaccines against SARS-CoV-2 infection. In addition, immunogenic peptides identified in COVID-19 sera could be used to determine the similarity of the immune recognition between SARS-CoV-2-infected and vaccinated individuals.

It is documented that antibody levels in response to SARS-CoV-2 infection decline over time (15, 16). This decline in antibody titer could contribute to COVID-19 reinfection (17). Ibarrondo et al. have reported that antibody titer declines rapidly with the half-life of 36 days in mild form cases of COVID-19 (18). Authors express concern about the duration of antibody responses to SARS-CoV-2 after infection and, as a result, the extent of lasting immunity following natural infection. Data on antibody response in COVID-19 are mainly based on the analysis of reactivity to whole S and N proteins and their peptides (12–14). This immune response analysis recognizes multiple epitopes across these proteins. However, the extent of lasting reactivity to specific peptides after infection remains largely unknown. By identifying peptides containing epitopes inducing long circulating antibodies, it may be possible to achieve better selection of strong and long-lasting targets for vaccination.

In the present study, we have further advanced our understanding of the biomarkers of fatal COVID-19 outcomes by examining serum reactivity with S and N protein peptides as well as cytokine activation. We show that in fatal cases, IgM reactivity is greater with N peptides than with S peptides but higher with S peptides in milder cases of COVID-19. Further, higher serum levels of pro-inflammatory cytokines were found in fatal COVID-19 cases. Among these cytokines, increased interleukin (IL)-18 and IL-6 appear to be the most significant observation, confirming the role of these cytokines in fatal COVID-19. Additionally, the increased serum level of chemokines and cytokines activating macrophages and neutrophils was demonstrated in fatal COVID-19 cases. We also observed differences in IgG reactivity between fatal and non-fatal COVID-19 sera. Additionally, the longitudinal analysis of IgG reactivity with SARS-CoV-2 S and N proteins identified peptides having the highest longevity in humoral immune response. We also identified S and N protein peptides and cytokines which could be used as early indicators of fatal COVID-19 outcomes.

## Materials and Methods

### Subjects

Acute serum samples were collected from 88 (70.8 ± 10.3 years old) COVID-19 patients (37 males and 51 females). Out of these acute samples, 62 and 26 samples were collected from non-fatal and fatal COVID-19, respectively. We also collected samples from 18 controls (65.3 ± 9.1 years old, 7 males and 11 females) which were age-matched to acute COVID-19. These age-matched control samples were used to analyze the acute serum data.

Additionally, 44 samples (37.7 ± 13.4 years old; 12 male and 32 female) were collected between 32 and 65 days (median days 42.0 ± 11.1; D42) and 32 serum samples (42.9 ± 13.5 years old; 8 male and 24 female) between 280 and 363 days (median days 306.0 ± 21.1; D306) after having positive SARS-CoV-2 RNA qPCR results and/or symptoms. D42 and D306 are herein referred to as early and late convalescent samples, respectively. To match the age of convalescent COVID-19 patients, serum samples from 27 controls were collected (47.1 ± 13.7 years old; 11 males, 16 females). This age-matched control group was used to analyze the convalescent data.

Clinical records were also collected for all patients. The diagnosis of COVID-19 was established based on clinical presentation and was confirmed by qPCR. All control serum samples were tested for anti-SARS-CoV-2 antibodies using the SARS-CoV-2 CoronaPass ELISA Kit (Genetico, Moscow, Russia). Only samples that are negative based on ELISA results were included as controls. Serum samples were stored at -80°C until used.

### Ethics Statement

The ethics committee of the Kazan Federal University approved this study, and signed informed consent was obtained from each patient and controls according to the guidelines adopted under this protocol (protocol 4/09 of the meeting of the ethics committee of the KSMA dated September 26, 2019). Sample collection in 2015–2016 was done according to a protocol approved by the Institutional Review Board of the Kazan Federal University, and informed consent was obtained from each respective subject according to the guidelines approved under this protocol (Article 20, Federal Law “Protection of Health Right of Citizens of Russian Federation” N323-FZ, 11.21.2011).

### COVID-19 Peptides

S and N protein peptides (20 aa) with 3-aa overlaps for SARS-CoV-2 were synthesized by Genscript (Jiangsu, China). SARS-CoV-2 S and N protein peptide aa sequences (purity >95%) are summarized in **Table 1**.

**Table 1:** Sequence and position of SARS-CoV-2 S and N protein peptides.

Peptide	aa sequence	Position	Peptide	aa sequence	Position			
Covid-N.1	MSDNGPQNQRNAPRITFGGP	1–20	Covid-N.10	NAAIVLQLPQGTTLPKGFYA	154–173	Covid-N.18	ELIRQGTDYKHWPQIAQFAP	290–309
Covid-N.2	GGPSDSTGSNQNGERSGARS	18–37	Covid-N.11	FYAEGSRGGSQASSRSSSRS	171–190	Covid-N.19	FAPSASAFFGMSRIGMEVTP	307–326
Covid-N.3	ARSKQRRPQGLPNNTASWF	35–54	Covid-N.12	SRSRNSSRNSTPGSSRGTSP	188–207	Covid-N.20	VTPSGTWLTYTGAIKLDDKD	324–343
Covid-N.4	WFTALTQHGKEDLKFPRGQG	52–71	Covid-N.13	TSPARMAGNGGDAALALLLL	205–224	Covid-N.21	DKDPNFKDQVILLNKHIDAY	341–360
Covid-N.5	GQGVPINTNSSPDDQIGYYR	69–88	Covid-N.14	LLLDRLNQLESKMSGKGQQQ	222–241	Covid-N.22	DAYKTFPPTEPKKDKKKKAD	358–377
Covid-N.6	YYRRATRRIRGGDGKMKDLS	86–105	Covid-N.15	QQQQGQTVTKKSAAEASKKP	239–258	Covid-N.23	KADETQALPQRQKKQQTVTL	375–394
Covid-N.7	DLSPRWYFYYLGTGPEAGLP	103–122	Covid-N.16	KKPRQKRTATKAYNVTQAFG	256–275	Covid-N.24	VTLLPAADLDDFSKQLQQSM	392–411
Covid-N.8	GLPYGANKDGIIWVATEGAL	120–139	Covid-N.17	AFGRRGPEQTQGNFGDQELI	273–292	Covid-N.25	LDDFSKQLQQSMSSADSTQA	409–428
Covid-N.9	GALNTPKDHIGTRNPANNAA	137–156						

### COVID-19 ELISA

The SARS-CoV-2-CoronaPass ELISA Kit (Genetico, Moscow, Russia) was used to determine SARS-CoV-2-specific antibodies IgM, IgG, and IgA according to the manufacturer’s instructions. The specificity and sensitivity of the SARS-CoV-2-CoronaPass ELISA Kit are 100% and 98.7%, respectively (19). Briefly, COVID-19 and control sera were mixed with conjugate-1 at a 1:10 ratio and incubated for 30 min at 37°C in a 96-well plate with pre-adsorbed SARS-CoV-2 antigens. Inactivated human serum without antibodies to SARS-CoV-2 served as a negative control (provided within the kit). Following washes (3×; 0.5% Tween 20 in PBS, PBS-T), wells were incubated with anti-human-IgG+IgM+IgA-HRP-conjugated antibodies for 30 min at 37°C. Post incubation and washes (3×; 0.5% Tween 20 in PBS), wells were incubated with 3,3′,5,5′-tetramethylbenzidine (Chema Medica, Moscow, Russia). The reaction was stopped by adding an equal amount of 10% phosphoric acid (TatKhimProduct, Kazan, Russia). Data were measured using a Tecan 200 microplate reader (Tecan, Switzerland) at OD_450_ with reference OD_650_. The result was considered as positive when the ratio of the tested sample OD_450_ to the negative control OD_450_+0.15 was greater than 1.

### Peptide Reactivity With Serum Antibodies

Several peptides were analyzed for reactivity with COVID-19 sera as well as controls. Peptide sequences are summarized in **Table 1**. Each peptide (1 μg/100 μl) was added into a 384-well plate and incubated at 4°C for 18 h. The washed plates were incubated with serum samples (1:100; 50 μl American Qualex Technologies, San Clemente, CA, USA) at 4°C for 18 h. Following washes [3×; 0.5% Tween 20 in PBS (PBS-T)], wells were incubated with anti-human-IgG-HRP-conjugated antibodies (1:10,000 in PBS-T, American Qualex Technologies, USA) for 30 min at 37°C. The washed (3×; 0.5% PBS-T) wells were incubated with 3,3′,5,5′-tetramethylbenzidine (Chema Medica, Moscow, Russia). The reaction was stopped by adding an equal amount of 10% phosphoric acid (TatKhimProduct, Kazan, Russia). Data were captured using a microplate reader Tecan 200 (Tecan, Switzerland) at OD_450_ with reference OD_650_.

### Multiplex Analysis

Serum cytokine levels were analyzed using the Bio-Plex (Bio-Rad, Hercules, CA, USA) multiplex magnetic bead-based antibody detection kit following the manufacturer’s instructions. The Bio-Plex Pro Human Cytokine 48-Plex Screening Panel (12007283, Bio-Rad, Hercules, USA) was used for detection of serum cytokines. Serum aliquots (50 μl) were analyzed with a minimum of 50 beads per analyte acquired. Median fluorescence intensities were collected using a MAGPIX analyzer (Luminex, Austin, TX, USA). Each sample was analyzed in triplicate. Data collected were analyzed with MasterPlex CT control software and MasterPlex QT analysis software (MiraiBio, San Bruno, CA, USA). Standard curves for each cytokine were generated using standards provided by the manufacturer.

### Statistical Analysis

Statistical analysis was performed in the R environment (20). Statistically significant differences between comparison groups were accepted as p < 0.05, assessed by the Kruskal–Wallis test with Benjamini–Hochberg (BH) adjustment for multiple comparisons. Correlations were analyzed using the R psych package (21) (based on Spearman’s rank correlation coefficient, p-values were adjusted with the Benjamini–Hochberg method).

## Results

### Clinical Presentation of COVID-19

There were 88 acute and 76 convalescent serum samples collected. The convalescent samples were split into early (median 42.0 ± 11.1 days) or late (median 306.0 ± 21.1 days) convalescence based on number of days after the first symptoms of COVID-19 and/or positive SARS-CoV-2 PCR test. Diagnosis of COVID-19 was established based on epidemiological anamnesis and clinical presentation and confirmed by SARS-CoV-2 qPCR analysis of nasopharyngeal swab. Clinical manifestation included mild (60 cases), moderate (51 cases), and severe (53 cases) forms. Out of 88 acute COVID-19 cases, 62 samples were from non-fatal and 26 samples were fatal COVID-19. These fatal cases had a severe form of COVID-19. The scale of lung damage of less than 20%, 20–40%, and more than 40% was found in 137, 23, and 4 patients, respectively. Fever was detected in all patients (37.92 ± 0.66°C) with a duration 6.31 ± 4.04 days. None of the COVID-19 convalescent patients required artificial ventilation or were hospitalized in an ICU.

### Analysis of S and N SARS-CoV-2 Peptide Reactivity in Acute COVID-19 Sera

**Figure 1:**
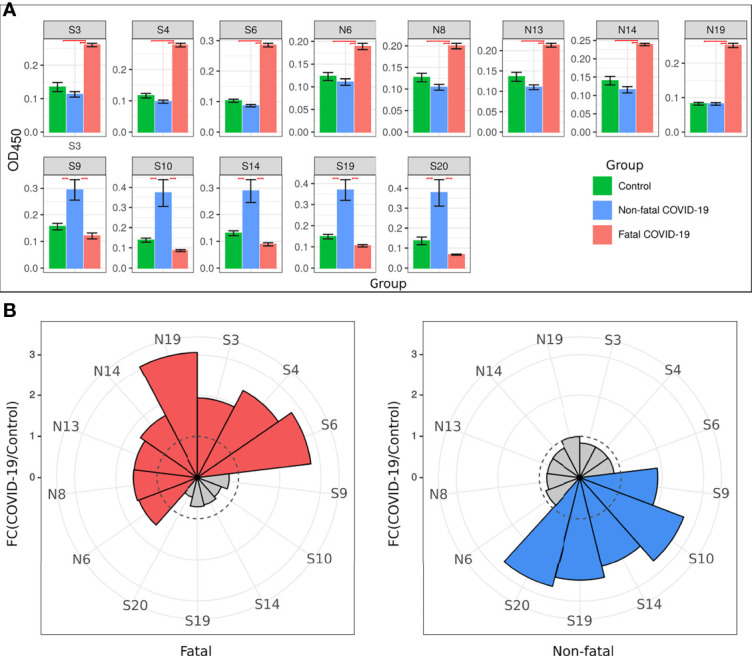
Serum IgM reactivity with S and N SARS-CoV-2 peptide in non-fatal and fatal COVID-19. Serum from acute COVID-19 was used to determine IgM reactivity with SARS-CoV-2 S and N protein peptides using ELISA. **(A)** Bar graph of serum reactivity with SARS-CoV-2 S and N peptides in non-fatal and fatal COVID-19. Data is presented as mean±SEM (standard error of mean). Red brackets indicate statistically significant differences (p < 0.05, Kruskal-Wallis test with BH adjustment). **(B)** Nightingale rose plots demonstrating SARS-CoV-2 S and N peptides differentially reactive with serum from non-fatal and fatal COVID-19 cases. Red and blue – statistically significant reactivity between COVID-19 and control samples in fatal and non-fatal COVID-19 cases, respectively (p < 0.05, Kruskal-Wallis test with BH adjustment); Grey – reactivity does differ significantly between COVID-19 and control samples. Data is presented as fold change – mean value of reactivity to peptide in COVID-19 sera divided by mean of reactivity to the same peptides in control sera.

Analysis of anti-SARS-CoV-2 IgM reactivity with S and N peptides revealed distinct patterns between cases of acute non-fatal and fatal COVID-19 (**Figure 1A**). COVID-19 serum reactivity was significantly increased with a total of eight S [S3 (p < 0.0001), S4 (p < 0.0001), S6 (p < 0.0001), S9 (p = 0.022), S10 (p = 0.046), S14 (p = 0.018), S19 (p = 0.018), and S20 (p = 0.028)] and five N peptides [N6 (p < 0.0001), N8 (p < 0.0001), N13 (p < 0.0001), N14 (p < 0.0001), and N19 (p < 0.0001)] compared to controls. However, when samples were analyzed based on patient outcome, reactivity with the five N peptides was only significantly higher [N6 (p < 0.0001), N8 (p < 0.0001), N13 (p < 0.0001), N14 (p < 0.0001), and N19 (p < 0.0001)] in cases of fatal COVID-19. These fatal cases also only showed higher reactivity with three of the S peptides [S3 (p < 0.0001), S4 (p < 0.0001), and S6 (p < 0.0001)]. In contrast, five S peptides (S9, S10, S14, S19, and S20) and none of the N peptides had increased reactivity with non-fatal COVID-19 serum compared with controls (**Figure 1B**).

**Figure 2:**
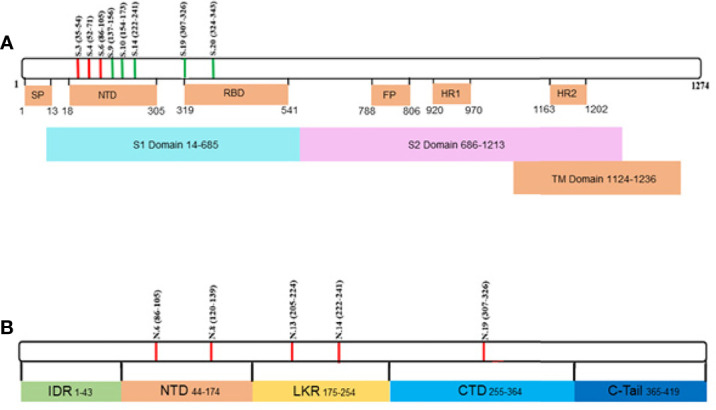
Schematic presentation of S and N protein peptides location reacting with non-fatal and fatal COVID-19. **(A)** Location of S protein peptides reacting with non-fatal and fatal COVID-19 IgM; **(B)** Location of N protein peptides reacting with fatal COVID-19 IgM. Red color – peptides reacting with fatal COVID-19 IgM; Green color – peptides reacting with non-fatal COVID-19 IgM. S1, Spike 1; S2, Spike 2; TM, Transmembrane; SP, Signal Peptide; NTD, N-terminal Domain; RBD, Receptor Binding Domain; FP, Fusion Peptide; HR1, Heptad Repeat 1; HR2, Heptad Repeat 2; IDR, Intrinsically Disordered Region; NDT, N-terminal Domain; LKR, Linked Region; CTD, C-terminal Domain.

Collectively, analysis of IgM revealed more frequent reactivity of acute fatal COVID-19 with N protein peptides, while non-fatal COVID-19 sera had more reactivity with S protein peptides. When the locations of the reactive peptides were analyzed, we found that all S peptides identified by acute fatal IgM were in the N-terminal domain (NTD) of the S protein (**Figure 2**). In contrast, S peptides highly reactive in non-fatal COVID-19 were located in the NTD and receptor-binding domain (RBD) (**Figure 2A**). Increased reactivity with N protein peptides was only found in fatal cases of COVID-19. These peptides were located in the NTD, linked region (LKR), and C-terminal domain (CTD) of N protein (**Figure 2B**).

### IgG Antibody Reactivity

**Figure 3:**
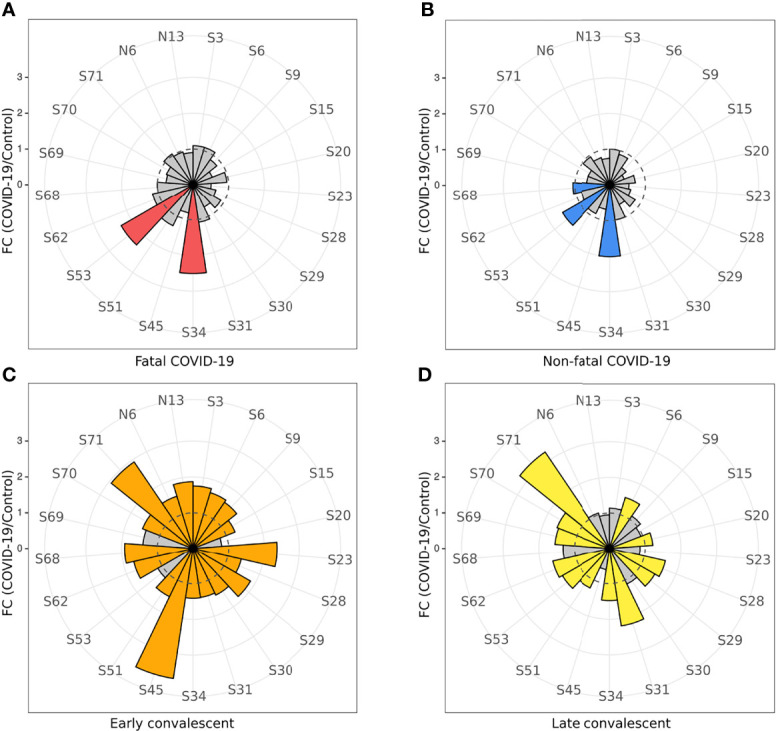
COVID-19 serum IgG reactivity with S and N SARS-CoV-2 peptides. Nightingale rose plots demonstrating reactivity of acute (non-fatal and fatal) COVID-19 and convalescent serum (early and late convalescent) with SARS-CoV-2 S and N peptides. IgG reactivity with SARS-CoV-2 S and N protein peptides was analyzed using ELISA. **(A)** IgM reactivity with S and N SARS-CoV-2 peptides in fatal COVID-19; **(B)** IgM reactivity with S and N SARS-CoV-2 peptides in non-fatal COVID-19; **(C)** IgM reactivity with S and N SARS-CoV-2 peptides in early convalescent COVID-19; **(D)** IgM reactivity with S and N SARS-CoV-2 peptides in late convalescent COVID-19. Red, blue, orange and yellow – statistically significant IgG reactivity in COVID-19 as compared to control (p < 0.05, Kruskal-Wallis test with BH adjustment). Data is presented as fold change – mean value of reactivity to peptide in COVID-19 divided by mean of reactivity to the same peptides in control.

Analysis of acute IgG reactivity with S and N peptides revealed a difference in S peptide reactivity between serum samples from fatal and non-fatal COVID-19 cases (**Figures 3A, B**). Fatal COVID-19 sera significantly reacted with S34 (p < 0.0001) and S53 (p < 0.0001), while non-fatal COVID-19 significantly reacted with S34 (p < 0.0001), S53 (p = 0.008), and S68 (p = 0.032) peptides. There was no reactivity of IgG with N peptides from both COVID-19 serum groups.

In contrast, multiple peptides of SARS-CoV-2 were found to be significantly reactive with convalescent COVID-19 IgG (**Figure 3**) compared to uninfected controls. Only three peptides were found to be significantly [S34 (p < 0.0001), S53 (p = 0.008), and S68 (p = 0.032) reactive in the acute non-fatal COVID-19 cases (**Figure 3B**), whereas more peptides (18 peptides) were found to be reactive in early convalescence (S3 (p = 0.007), S6 (p = 0.018), S9 (p = 0.042), S15 (p = 0.014), S23 (p = 0.0005), S28 (p = 0.034), S29 (p < 0.0001), S30 (p = 0.014), S31 (p = 0.0008), S34 (p = 0.038), S45 (p < 0.0001), S51 (p = 0.007), S62 (p = 0.0001), S68 (p = 0.018), S70 (p = 0.013), S71 (p < 0.0001), N6 (p = 0.042), and N13 (p = 0.033)] and late convalescence (12 peptides) [S6 (p = 0.007), S20 (p < 0.0001), S28 (p = 0.002), S29 (p = 0.001), S31 (p < 0.0001), S34 (p = 0.001), S51 (p = 0.020), S53 (p = 0.0008), S62 (p < 0.0001), S69 (p = 0.0006), S70 (p = 0.0007), and S71 (p < 0.0001)] when compared to controls. Three features of the convalescent serum reactivity were recognized; firstly, more peptides were reactive following recovery compared to the acute COVID-19 stages (**Figures 3B, C**). Secondly, peptides S34, S53, and S68 were consistently significantly reactive during acute and either the early or late convalescent COVID-19 when compared to controls (**Figures 3B, C**). Additionally, peptides S6 (p = 0.018, p = 0.007), S28 (p = 0.034, p = 0.002), S29 (p < 0.0001, p = 0.001), S31 (p = 0.0008, p < 0.0001), S34 (p = 0.038, p = 0.001), S51 (p = 0.007, p = 0.020), S62 (p = 0.0001, p < 0.0001), S70 (p = 0.013, p = 0.0007), and S71 (p < 0.0001, p < 0.0001) were significantly reactive in the early and late convalescent COVID-19 stages when compared to controls (**Figures 3C, D**). Some peptides remained reactive up to 12 months postinfection with 12 peptides showing increased reactivity with late convalescence serum in contrast to only three peptides in acute serum samples (**Figures 3B, D**). Finally, the number of reactive peptides declined with months postinfection with 18 peptides in early convalescence samples vs. 12 in late convalescent samples (**Figures 3B, D**). Interestingly, at the early convalescent phase, two N protein peptides (N6 (p = 0.042) and N13 (p = 0.033) were significantly reactive with COVID-19 IgG, while reactivity to N proteins was absent in late convalescence as compared to controls.

**Figure 4:**
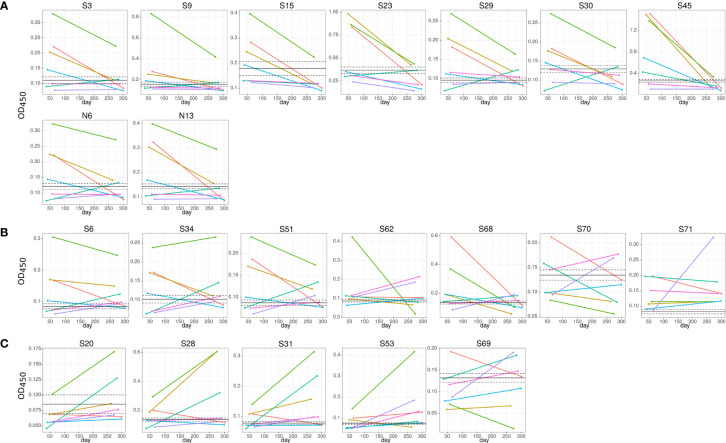
Dynamics of convalescent COVID-19 IgG antibody reactivity with SARS-CoV-2 S protein peptides. Serum from early (median 42.0±11.1) and late (median 306.0±21.1) convalescent COVID-19 was used for analysis. IgG reactivity with SARS-CoV-2 S protein peptides was analyzed using ELISA. **(A)** IgG reactivity decreased in 5 out of 7 COVID-19 convalescent serum with time post infection; **(B)** reactivity with peptides remained mostly unchanged; **(C)** IgG reactivity increased in 5 out of 7 COVID-19 convalescent serum with time post infection. Lines represent individual COVID-19 convalescent sample. S6, S15, S20, S31, S34 and S45 – are SARS-CoV-2 S protein peptides.

We have also found a difference in the dynamics of reactivity with SARS-CoV-2 S and N peptides (**Figure 4** and **Table 2**). There were three groups of peptides identified based on longevity of the reactivity with SAR-CoV-2 peptides. Group 1 contained peptides with which reactivity with COVID-19 convalescent serum declined between early and late convalescence. The peptide with the greatest decline in reactivity from early to late convalescence was S45 (**Figure 4A**). Other peptides possessing declined reactivity were S3, S9, S15, S23, S29, S30, N6, and N13. Group 2 included peptides whose reactivity with COVID-19 convalescent serum remained mostly unchanged (S6, S34, S51, S62, S68, S70, and S71) (**Figure 4B**). Peptides in group 3 were more reactive in late compared to early convalescence samples. These peptides were S20, S28, S31, S53, and S69 (**Figure 4C**).

**Table 2:** Analysis of longitudinal reactivity of COVID-19 serum with SARS-CoV-2 S and N protein peptides.

	Peptide	COVID-19 (D42)		COVID-19 (D306)	
		N	%	N	%
Group 1	S3	31/44	70.45	14/42	33.33
	S9	27/44	61.36	17/42	40.48
	S15	22/44	50.00	9/42	21.43
	S23	35/44	79.55	8/42	19.05
	S30	31/44	70.45	18/42	42.86
	S45	36/44	81.82	3/42	7.14
	N6	30/44	68.18	14/42	33.33
	N13	31/44	70.45	12/42	28.57
Group 2	S6	30/44	68.18	29/42	69.05
	S28	27/44	61.36	28/42	66.67
	S29	36/44	81.82	30/42	71.43
	S31	30/44	68.18	33/42	78.57
	S34	25/44	56.82	28/42	66.67
	S47	21/44	47.73	24/42	57.14
	S51	30/44	68.18	24/42	57.14
	S68	28/44	63.64	25/42	59.52
	S20	3/44	6.82	15/42	35.71
Group 3	S53	22/44	50.00	31/42	73.81
	S62	33/44	75.00	36/42	85.71
	S69	26/44	59.09	33/42	78.57
	S70	29/44	65.91	32/42	76.19
	S71	38/44	86.36	42/42	100.00

n, number of COVID-19 convalescent serum samples with SARS-CoV-2 S and N peptides higher than the mean ± SE in control; %, percent COVID-19 convalescent serum samples with reactivity higher than the mean ± SE in control.

**Figure 5:**
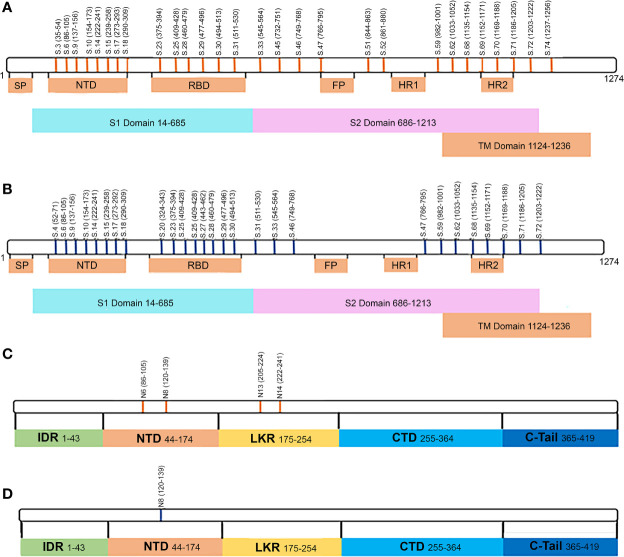
Schematic presentation of S and N protein peptide locations that are reactive with fatal and non-fatal COVID-19 sera. **(A)** Location of S protein peptides reacting with IgG serum from early convalescent COVID-19; **(B)** Location of N protein peptides reacting with IgG serum from early convalescent COVID-19; **(C)** Location of S protein peptides reacting with IgG serum from late convalescent COVID-19; **(D)** Location of N protein peptides reacting with IgG serum from late convalescent COVID-19; Orange color – peptides reactive with early (1-2 months) convalescent COVID-19 IgG sera; Blue color – peptides reacting with late (10-12 months) convalescent COVID-19 IgG sera.

Peptides reacting with early and late convalescent serum samples were mapped to different domains of the S protein (**Figure 5**). We found that reactivity of peptides in the N-terminal domain (NTD), receptor-binding domain (RBD), and heptad repeat 2 (HR2) was high in both early and late convalescence samples, whereas peptides in the Spike 2 (S2) domain, namely, the fusion peptide (FP), were only highly reactive in early convalescence samples. We also examined the location of the N protein peptides with high reactivity in convalescent samples. Reactivity of peptides in the NTD and linker region (LKR) of the N protein was found in early convalescence samples but only located in the NTD in late convalescence samples. These data suggest that during the convalescent phase, there are still antibodies circulating, which could have a potential to neutralize the virus.

### Serum Cytokine Analysis in COVID-19

**Figure 6:**
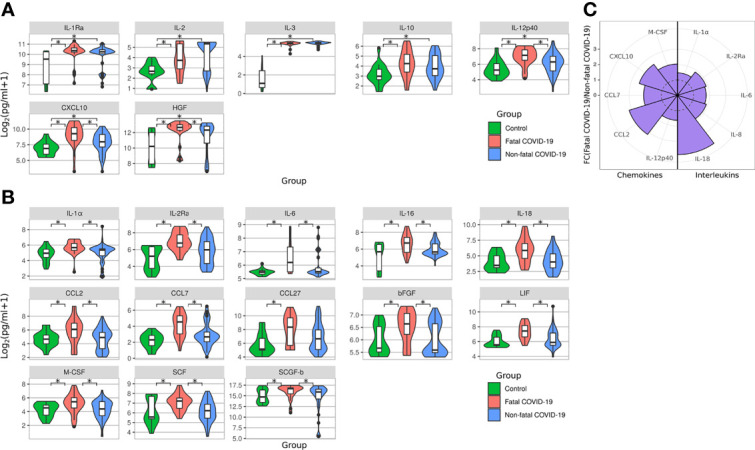
Serum cytokine level in fatal and survived COVID-19. Violine plot demonstrating serum cytokines level in acute COVID-19 analyzed using Bio-Plex (Bio-Rad, Hercules, CA, USA) multiplex magnetic bead-based antibody detection kit. **(A)** Cytokines upregulated in non-fatal and fatal COVID-19 compared to controls; **(B)** Cytokines upregulated only in fatal COVID-19 compared to controls; Data is presented as violin plots with boxplots of Log2 of cytokines concentration.*p < 0.05 (Kruskal-Wallis test with BH adjustment). **(C)** Nightingale rose plots demonstrating serum cytokine level in non-fatal and fatal COVID-19 using the Bio-Plex (Bio-Rad, Hercules, CA, USA) multiplex magnetic bead-based antibody detection kit. Purple –increased reactivity in fatal COVID-19 compared to non-fatal COVID-19 samples (p < 0.05, Kruskal-Wallis test with BH adjustment). Dotted line – fold change = 1. Data is presented as fold change – mean value of cytokines in fatal COVID-19 divided by mean of cytokines in non-fatal COVID-19.

We analyzed serum levels of cytokines in cases of fatal and non-fatal COVID-19. We first observed a significantly elevated level of 7 serum cytokines (IL-1Ra, IL-2, IL-3, IL-10, IL-12p40, CXCL10, and HGF) in all COVID-19 cases when compared with controls (**Figure 6A**). Of these cytokines, a greater number [20 cytokines: IL-1Ra (p = 0.0006), IL-1α (p = 0.003), IL-2 (p = 0.038), IL-2Ra (p = 0.0001), IL-3 (p < 0.0001), IL-6 (p = 0.002), IL-10 (p = 0.006), IL-12p40 (p < 0.0001), IL-16 (p = 0.008), IL-18 (p = 0.001), CCL2 (p = 0.017), CCL7 (p < 0.0001), CCL27 (p = 0.004), CXCL10 (p < 0.0001), bFGF (p = 0.031), HGF (p = 0.0001), LIF (p = 0.0002), M-CSF (p = 0.030), SCF (p = 0.028), and SCGF-b (p = 0.029)] were significantly elevated in fatal cases than in non-fatal cases [7 cytokines; IL-1Ra (p = 0.006), IL-2 (p = 0.0003), IL-3 (p < 0.0001), IL-10 (p = 0.014), IL-12p40 (p = 0.022), CXCL10 (p = 0.01)] (**Figures 6A, B**). We also compared the levels of cytokines in fatal to those in non-fatal cases. There were 15 cytokines [IL-1α (p = 0.002), IL-2Ra (p = 0.0008), IL-6 (p = 0.004), IL-12p40 (p = 0.004), IL-16 (p = 0.008), IL-18 (p = 0.0008), CCL2 (p = 0.01), CCL7 (p = 0.001), CXCL10 (p = 0.002), bFGF (p = 0.01), HGF (p = 0.020), LIF (p = 0.002), M-CSF (p = 0.019), SCF (p = 0.002), and SCGF-b (p = 0.049)] with significantly higher levels in fatal cases compared with non-fatal cases (**Figures 6A, B**).

As expected, significantly increased activation of pro-inflammatory cytokines (IL-1α, IL-2Ra, IL-6, IL-8, and IL-18) in fatal COVID-19 compared to non-fatal COVID-19 sera was measured (**Figure 6C**). Additionally, the level of multiple chemokines (IL-12p40, CCL2, CCL7, CXCL10, and M-CSF) was significantly increased in fatal COVID-19 cases. These data support previous evidence that highly elevated cytokines and the “cytokine storm” contribute to fatal COVID-19 pathogenesis (22–24).

### Diagnostic Value of Peptide Reactivity and Cytokine Activation

**Figure 7:**
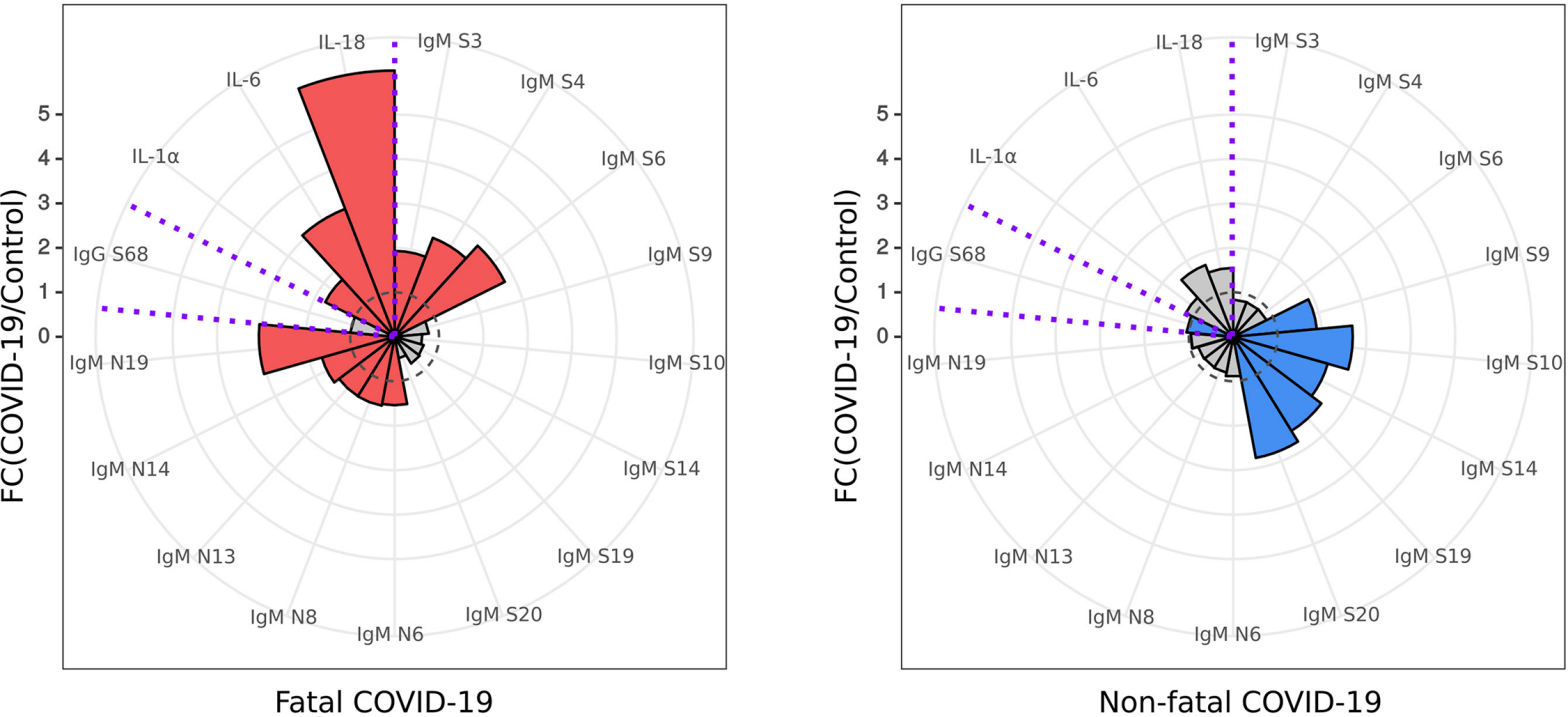
Diagnostic panel for early identification of fatal COVID-19. Serum cytokine (IL-1α, IL-6 and IL-18) level and reactivity of anti-SARS-CoV-2 IgM and IgG with S and N protein peptides selected for a diagnostic panel for early identification of fatal COVID-19. Red – cytokine level and SARS-CoV-2 peptide reactivity in fatal COVID-19; Blue – cytokine level and SARS-CoV-2 peptide reactivity in non-fatal COVID-19. Dotted line – fold change = 1, level in control. Data is presented as fold change – mean value of cytokines in COVID-19 divided by mean of cytokines in control.

Using the data presented here on IgM SARS-CoV-2 peptide reactivity and serum cytokine levels of IL-1α, IL-6, and IL-18, we have identified a unique biomarker panel which could be used for early identification of COVID-19 patients with increased risk of severe and potentially fatal disease (**Figure 7**).

## Discussion

Distinct immune responses and patterns of cytokine activation previously documented have uncovered several biomarkers associated with COVID-19 severity (25–27). Our data provide a more comprehensive picture and significantly advance the current understanding about the humoral immune response in fatal and non-fatal COVID-19 cases through identification of a distinct pattern of antibody recognition of S and N protein peptides. The most striking difference was a lack of IgM antibody reactivity with N protein peptides in non-fatal patients. Also, we report that the panels of S protein peptides reacting with fatal and non-fatal COVID-19 differ. Specifically, only non-fatal COVID-19 sera had reactive peptides located in the RBD of S protein. Importantly, the RBD is one of the targets for neutralizing antibodies (28) and anti-RBD antibody levels have been shown to correlate with neutralizing activity (18). Our analysis revealed that S20, a peptide exclusively reacting with non-fatal acute IgM, contains neutralizing epitopes identified by Barnes et al. (28), thus supporting previous observations that development of neutralizing antibodies is delayed in fatal COVID-19 compared to non-fatal COVID-19 cases (29). In addition to the RBD, the NTD can be targeted by neutralizing antibodies (30), although there is higher potency of RBD-recognizing antibodies demonstrated by Graham et al. (31). Therefore, we suggest that a larger number of peptides recognized by survivor IgM antibodies on the RBD and the NTD regions contribute to convalescence.

Evidence shows that the immune response to SARS-CoV-2 infection contributes to COVID-19 outcomes (32, 33). Reactivity to S and N proteins appears to differ between non-fatal and fatal cases (34); however, little is known about the location of immunogenic regions in these proteins. We identified multiple N protein peptides reacting with fatal COVID-19 IgM. These peptides were located in the NTD and LKR and C-terminal domain (CTD) regions of the N protein. These regions were previously shown to contain immunogenic epitopes (35–37). Similarly, Heffron et al. identified N protein peptides located in the CTD which highly correlated with intubated patients, when compared with non-hospitalized patients (37). Multiple epitopes in the NTD and LKR regions have also been identified as reacting with severe COVID-19 patient sera (14). This commonly observed reactivity to N protein in severe and fatal patients suggests that early screening for the presence of anti-N protein antibodies could be a prognostic factor for clinical outcome, helping to identify patients for high risk of developing severe and fatal COVID-19 during admission (38). The role of anti-N protein antibodies in pathogenesis of severe COVID-19 remains largely unknown. Recently, Batra et al. have suggested that SARS-CoV-2 N protein could contribute to the severity of the disease by inducing non-neutralizing antibodies with the ability to induce an antibody-dependent enhancement (ADE) response (38). This assumption is supported by the high homology between N protein from SARS-CoV-2 and other coronaviruses (38). It was suggested that previous exposure could lead to the circulation of the large quantity of cross-reacting anti-coronavirus N protein antibodies capable of ADE (38, 39).

We have also found that peptides recognized during early and late convalescence differ following two major trends: firstly, the number of reactive peptides declined with time post convalescence, and secondly, the overall intensity of antibody binding to peptides declined from early to late convalescence. These data corroborate previous observations that the humoral immune response declines with time post recovery (15, 40). Substantial reduction in the number of peptides and reaction intensity to NTD and RBD peptides of the S protein was found in late convalescence. Similarly, reactivity to N peptides was reduced as time passed such that there was no reactivity to these peptides by 306 days after recovery. These data are in agreement with previous reports showing that anti-S protein IgG levels remained elevated for longer compared to anti-N protein IgG levels (41, 42). Therefore, it could be suggested that anti-S protein antibodies are the optimal markers of an anti-SARS-CoV-2 immune response.

Changes in serum cytokine levels were also examined as these factors were identified early during the pandemic outbreak in playing a central role in COVID-19 pathogenesis (27). The “cytokine storm” and its major contributor IL-6 (43) have been highlighted as potential therapeutic targets (44). We have identified multiple cytokines known to induce and maintain inflammation as activated in fatal but not non-fatal COVID-19 cases. Among these cytokines was IL-6, confirming previous observations of its role in severe COVID-19 pathogenesis (45). Additionally, we found an increased level of M-CSF in fatal COVID-19 but not non-fatal COVID-19 sera. This inflammatory mediator has overlapping functions with GM-CSF, another cytokine previously identified as being highly upregulated in fatal COVID-19 (46). The “cytokine storm” hypothesis is further supported by our findings given that an increased level of two powerful pro-inflammatory cytokines, IL-1α and IL-18, were found in fatal, not in non-fatal, COVID-19. These are IL-1 family cytokines with distinct functions. IL-1α is a principal cytokine maintaining inflammatory moiety in necrotic tissue (23). Therefore, a substantial increase in the level of this cytokine could indicate necrosis in COVID-19 patients. IL-18 is also a pro-inflammatory cytokine, produced by activated inflammasomes (47). This cytokine is released by activated macrophages and synergizes with IL-12 to activate T cell immune response which can induce fatal inflammation through activation of natural killer (NK) cells (22, 48, 49).

In addition to pro-inflammatory cytokines, we have found an increased level of multiple chemokines capable of attracting activated leukocytes to the site of infection. These chemokines, CCL2, CCL7, CCL27, and CXCL10, were shown to stimulate chemotaxis of monocytes, CD8 T cells, and NK cells which were identified as infiltrating tissues in COVID-19 (50, 51). Our data also confirm the role of CCL2 and CXCL10 in severe COVID-19 as these chemokines were found to be increased in serum of patients admitted to ICU (46, 52). Additionally, our data further support the hypothesis of dysregulation of mononuclear phagocytes (52, 53), as CCL2, increased in COVID-19, promotes macrophage migration and differentiation (54). The role of neutrophils in the pathogenesis of fatal COVID-19 could also be suggested as CCL7 contributes to the accumulation of these granulocytes in the lung (55). Interestingly, Xie et al. (52) showed that neutralization of CCL7 attenuated angiotensin II-induced macrophage infiltration. This role of macrophages in pathogenesis of COVID-19 is supported by an increased level of M-CSF found in fatal cases. Together, excessive M-CSF-driven monocyte/macrophage proliferation and CCL2/CCL7 activation and chemotaxis could be the mechanism of severe and fatal COVID-19 pathogenesis.

Levels of IL-1β were not affected, while, in contrast, serum IL-18 was increased in fatal COVID-19 cases. A previous study using an animal model of acute respiratory distress syndrome (ARDS), that is frequently diagnosed in critical COVID-19 cases (56), demonstrated that serum levels of IL-18 could serve as a biomarker of severity and mortality (57). A similar conclusion was presented by Satis et al., who showed that higher levels of IL-18 were found in serum of COVID-19 with worse outcomes (58).

We have identified SARS-CoV-2 S and N peptides that can be used for early prediction of fatal COVID-19 outcomes. Our data confirm that reactivity with N protein peptides is more prevalent in fatal than non-fatal COVID-19 sera. Additionally, we have found higher levels of pro-inflammatory cytokines and chemokines in fatal COVID-19 sera, supporting the role of “cytokine storm” in the pathogenesis of severe COVID-19. Among these cytokines, IL-18 appears to have a special role as it can be released by activated macrophages and neutrophils and, thus, combined with IL-12, could contribute to COVID-19 fatality. Higher levels of CCL2 and CCL7 chemokines as well as M-CSF also implicate the role of macrophages and neutrophils in pathogenesis of cytokine storm. From these data on S and N protein peptide reactivity and cytokine activation, we provide a panel of clinically significant biomarkers which could be used for early prediction of COVID-19 fatality.

In conclusion, we have identified several markers that could be used for the early prediction of fatal COVID-19 outcomes. We also confirm the prediction value of antibody reactivity with SARS-CoV-2 N protein and the high serum levels of IL-6 in COVID-19 patients. Moreover, we have identified novel markers, including N and S protein peptides, that are reactive in the case of fatal COVID-19. Higher levels of IL-1α and IL-18 pro-inflammatory cytokines were also found in fatal COVID-19 serum. Using these novel markers, we have developed a panel of biomarkers that could be used for the early prediction of COVID-19 fatality risk.

## Data Availability Statement

The original contributions presented in the study are included in the article/supplementary material. Further inquiries can be directed to the corresponding authors.

## Ethics Statement

The ethics committee of the Kazan Federal University approved this study, and signed informed consent was obtained from each patient and controls according to the guidelines adopted under this protocol (protocol 4/09 of the meeting of the ethics committee of the KSMA dated September 26, 2019). Sample collection in 2015–2016 was done according to a protocol approved by the Institutional Review Board of the Kazan Federal University, and informed consent was obtained from each respective subject according to the guidelines approved under this protocol (Article 20, Federal Law “Protection of Health Right of Citizens of Russian Federation” N323-FZ, 11.21.2011). The patients/participants provided their written informed consent to participate in this study.

## Author Contributions

EM, SH, EG, VS, NK, and MB designed and performed the experiments. EM, SH, MM, EG, YD, VS, NK, MB, RS-M, TF, and SK analyzed the data. All authors contributed to the writing of the manuscript. All authors contributed to the article and approved the submitted version.

## Funding

This study was supported by the Kazan Federal University Strategic Academic Leadership Program and by the subsidy allocated to Kazan Federal University for the state assignment in science (project #0671-2020-0058). Also, this work was supported by the Kazan Federal University Strategic Academic Leadership Program (PRIORITY-2030).

## Conflict of Interest

The authors declare that the research was conducted in the absence of any commercial or financial relationships that could be construed as a potential conflict of interest.

## Publisher’s Note

All claims expressed in this article are solely those of the authors and do not necessarily represent those of their affiliated organizations, or those of the publisher, the editors and the reviewers. Any product that may be evaluated in this article, or claim that may be made by its manufacturer, is not guaranteed or endorsed by the publisher.
